# Supplementary material for: Superconductivity and magnetism in iron sulfides intercalated by metal hydroxides
Source: Chem Sci. 2017 Mar 13;8(5):3781–8. doi: 10.1039/c6sc05268a (PMC5436553; doi:10.1039/c6sc05268a)
Supplement: Supplementary file 1 [file SC-008-C6SC05268A-s001.pdf]

Electronic Supplementary Material (ESI) for Chemical Science.
This journal is © The Royal Society of Chemistry 2017

Superconductivity in iron sulfides intercalated by metal hydroxides

Xiuquan Zhou[a,b], Christopher Eckberg[b,c], Brandon Wilfong[a,b], Sz-Chian Liou[d], Hector K. Vivanco[a], Johnpierre Paglione[b,c] and Efrain E. Rodriguez *[a,b]

[a]Department of Chemistry and Biochemistry, University of Maryland, College Park, MD 20742
[b]Center for Nanophysics and Advanced Materials, University of Maryland, College Park, MD 20742
[c]Department of Physics, University of Maryland, College Park, MD 20742
[d]AIM Lab, Maryland NanoCenter, University of Maryland, College Park, MD 20742

Supplementary Information

Diffraction, transport, magnetization and other characterization measurements

Powder X-ray diffraction (XRD) data were collected using a Bruker D8 X-ray diffractometer with Cu Kα radiation, $\lambda = 1.5418$ Å. High-resolution synchrotron X-ray diffraction were carried out at Beamline 11-BM at the Advanced Photon Source (APS). Diffraction data were collected between 0.5° and 46° with a step size of 0.0001° using a constant wavelength $\lambda = 0.414164$ Å (30 keV). Rietveld and Pawley refinements were carried out using TOPAS software.[1] Microscopic images were examined on a Hitachi SU-70 SEM field emission scanning electron microscope (SEM), and their elemental compositions were determined by energy dispersive X-ray spectroscopy (EDS) using a BRUKER EDS detector. Electron diffraction patterns were obtained using a JEM 2100 LaB_6 transmission electron microscope (TEM) at an acceleration voltage of 200 KeV.

Inductively coupled plasma atomic emission spectroscopy (ICP-AES) data were collected using an Shimadzu ICPE-9000 spectrometer. Standards used for ICP-AES were diluted from 1000 ppm of respective elements purchased from Sigma-Aldrich.

*efrain@umd.edu

Magnetic susceptibility measurements were performed using a Quantum Design Magnetic Properties Measurement System (MPMS). The volume fractions of superconducting phases were calculated based on the density obtained from Reitveld refinement. Electrical resistivity and heat capacity measurements were performed on a 14 T Quantum Design Physical Properties Measurement System (PPMS).

More notes on the $K_xFe_{2-y}S_2$ phase

While we did not find superconducting phases containing potassium, we did demonstrate that the synthetic temperature for the preparation of $K_xFe_{2-y}S_2$ can be lowered from about 1000 °C to 160 °C through hydrothermal methods. Without KOH, single crystals of $K_xFe_{2-y}S_2$ can be completely converted to mackinawite FeS. Therefore, the conversion between $K_xFe_{2-y}S_2$ and tetragonal FeS is fully reversible, as traced by the equilibrium reaction between **1** and **2** in Fig. 1. With further work on reducing the iron vacancies, the potassium intercalated phases could be made superconducting. To confirm this, we started to apply this route to the selenide system without optimization, and $K_xFe_{2-y}Se_2$ was prepared despite the presence of tetragonal FeSe. The implication of these results are that this hydrothermal route can lead to pure 122 type of layered compounds or the corresponding deintercalated tetragonal system. In addition, this hydrothermal route can be advantageous over solid-state route to avoid high temperature impurity phases or targeting compounds not thermodynamically stable at low temperature.

More notes on the structure of Na-Tochinilite

A projection of Fe atoms on the (001) plane in Na-tochilinite is illustrated in Fig. S8, and compared to perfect square lattice in tetragonal FeS, there is a clear distortion along the *b*-axis.

The comparison between the morphologies of $(Li_{1-x}Fe_xOH)FeS$ and NaOH intercalated FeS systems may provide further evidence of the hexagonal hydroxide layers with the NaOH reactions. Our $(Li_{1-x}Fe_xOH)FeS$ samples consisted mainly of square-shaped platelets in micron size (Fig. S10a and Fig. S10b), which would be indicative of the underpinning layered tetragonal structure. However, a similar morphology was not observed for either the *inc*-Na-tochinilite or Na-tochinilite samples(Fig.S10c and Fig. S10d, respectively). While still layered, the crystallites display irregular shapes instead of square platelets. The square-shaped platelets are consistent with the crystal habit of the tetragonal LiOH-intercalated FeS system.

References

[1] R. W. Cheary and A. Coelho, *J. Appl. Crystallogr.*, 1992, **25**, 109–121.

Table S1: List of $Li_{1-x}Fe_xOH)FeS$ samples. Detailed synthetic conditions are described in the above text, and only temperature, the most important factor, is shown in the table. Lattice constants of only representative samples are shown for duplicate samples. Because Na-tochilinite can be produced with the presence of NaOH, $Na_2S \cdot 9H_2O$ was not used as a precursor for powder samples due to its hydrolysis to NaOH and NaSH in solution. Li_2S was the main sulfur source used for powder samples, and every sample prepared with Li_2S has been reproduced at least once. Single crystal samples are not very homogeneous, and their T_c's vary from crystal to crystal, but their superconductivity is highly reproducible. Multiple single crystal batches have been reproduced at 120 °C suing different sulfur sources with the presence of Sn.

	**No.**	**Temperature (°C)**	**Sulfur source**	Sn (Y/N)	T_c **(K)**	a **(Å)**	c **(Å)**
	1	130	Li_2S	N	N/A	3.706	8.862
	2	160	Li_2S	N	N/A	3.704	8.942
	3	160	thiourea	N	N/A	3.696	8.979
	4	180	thiourea	N	N/A	3.702	8.943
Powder	5	200	thiourea	N	N/A	3.702	8.970
	6	120	thiourea	Y	2-3	3.700	8.919
	7	120	Li_2S	Y	2-3	3.704	8.900
	8	140	Li_2S	Y	2-3	3.706	8.900
	9	160	Li_2S	Y	2-3	3.704	8.888
	10	200	Li_2S	Y	N/A	3.701	8.926
	11	120	thiourea	Y	2-8	3.703	8.935
SC	12	120	$Na_2S \cdot 9H_2O$	Y	2-6	3.712	8.877
	13	120	Li_2S	Y	2-4	3.703	8.960

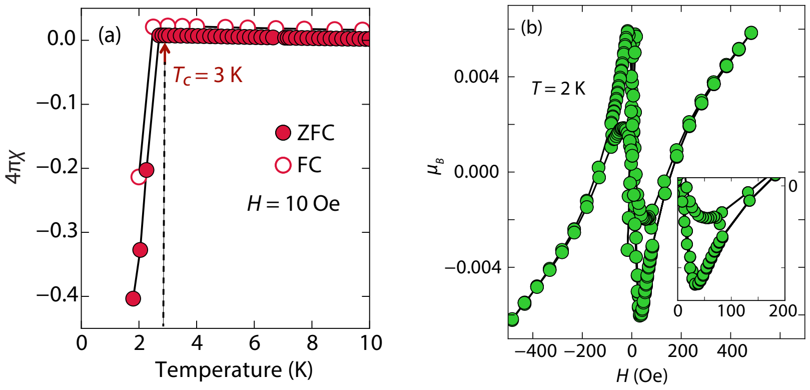

Figure S1: Magnetic susceptibility measurements of superconducting $(Li_{1-x}Fe_xOH)FeS$ at (a) constant field and (b) constant temperature. The H_{c1} and H_{c2} of this sample are about 40 and 180 Oe, respectively. The XRD pattern of this sample is shown in Fig. 3a

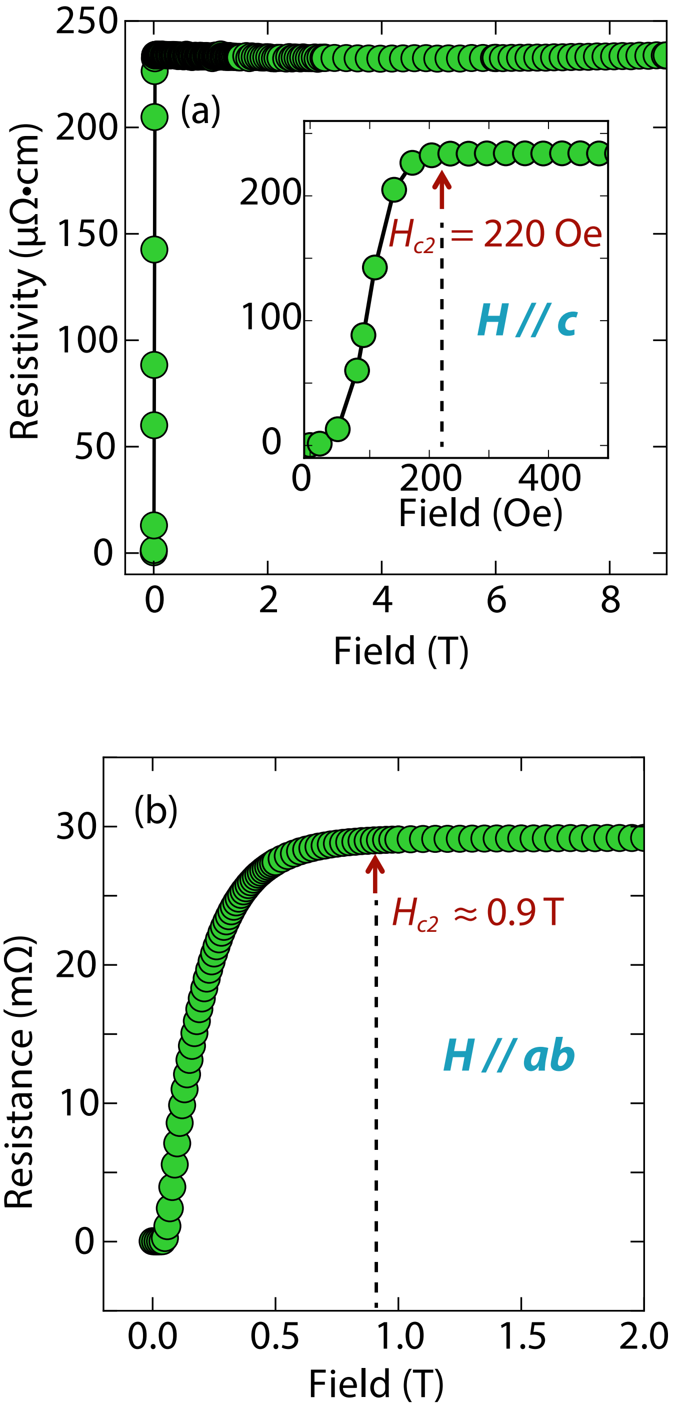

Figure S2: Field dependence of electrical resistance for a superconducting $(Li_{1-x}Fe_xOH)FeS$ sample ($T_c = 3.5$ K) at 1.8 K. The anisotropy of H//c and H//ab are shown in (a) and (b), respectively. Its temperature dependent electrical resistivity is shown in Fig. 2a.

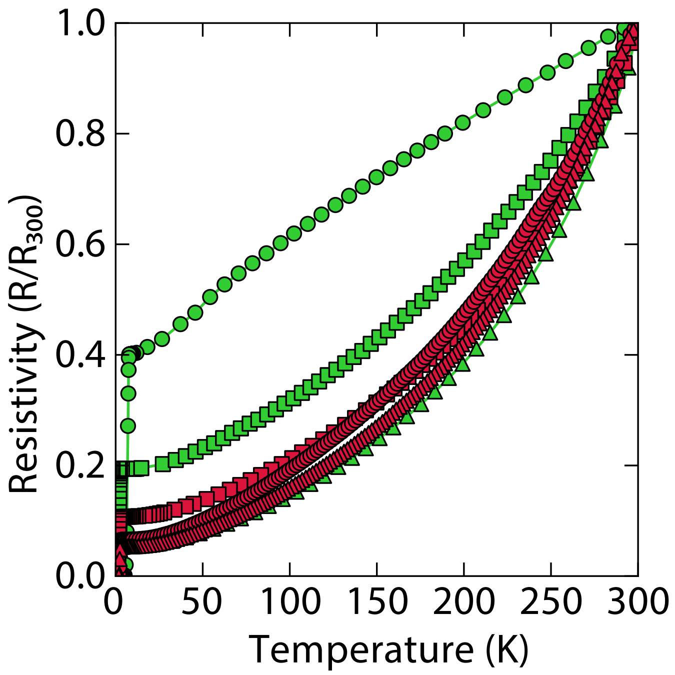

Figure S3: Temperature dependence of electrical resistivity for superconducting $(Li_{1-x}Fe_xOH)FeS$ samples. Green and red colors indicate samples prepared using thiourea and $Na_2S \cdot 9H_2O$, respectively.

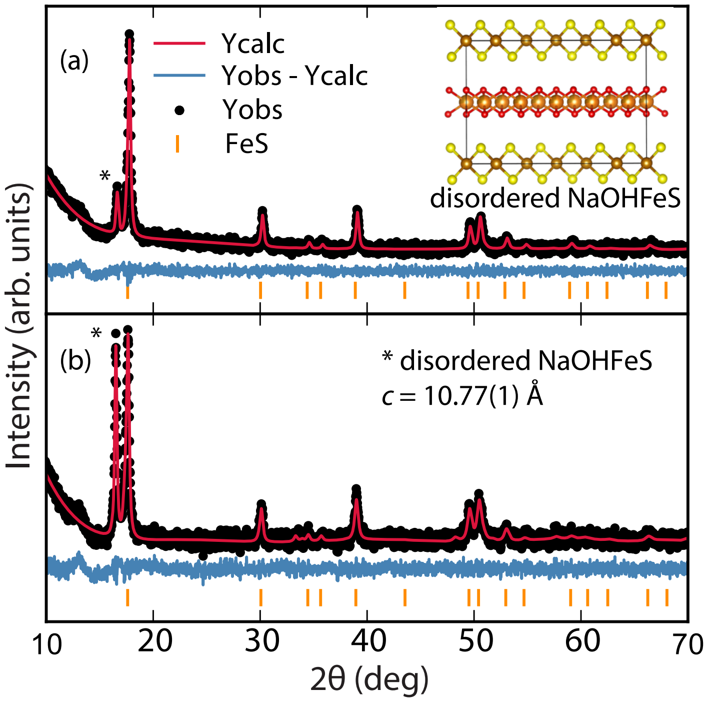

Figure S4: XRD patterns of mixtures of disordered NaOH intercalated FeS (indicated by *) and tetragonal FeS (indicated by tick marks) with significantly more tetragonal FeS in (a) than (b). The magnetic susceptibility of (a) and (b) are shown in Fig. S5 and Fig. 4, respectively.

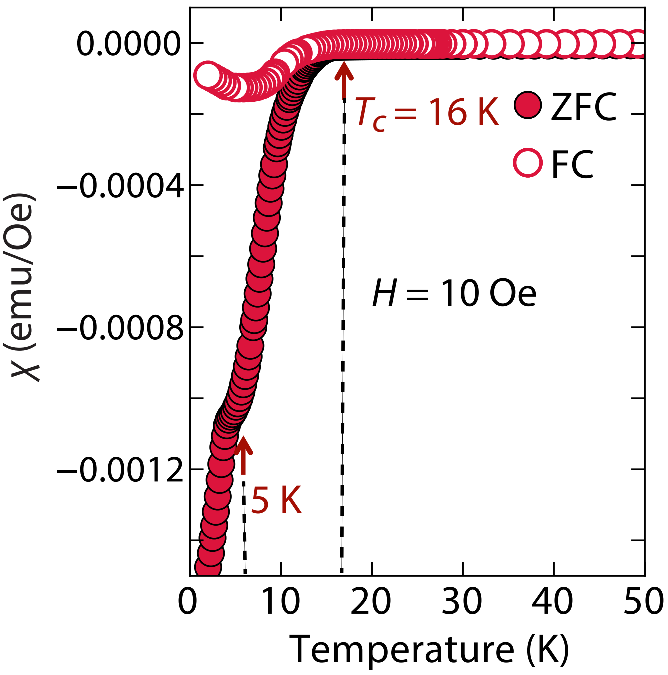

Figure S5: Temperature dependent magnetic susceptibility measurement of *inc*-Na-tochinilite with tetragonal FeS as a major phase. Its XRD pattern is shown in Fig. S4a

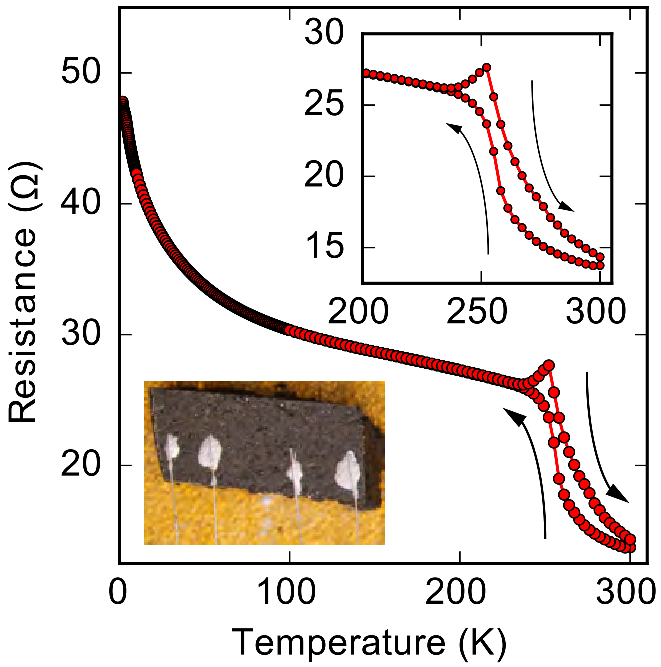

Figure S6: Electrical resistance of *inc*-Na-tochilinite as a function of temperature. The measurement was carried out on a pressed pellet from powders.

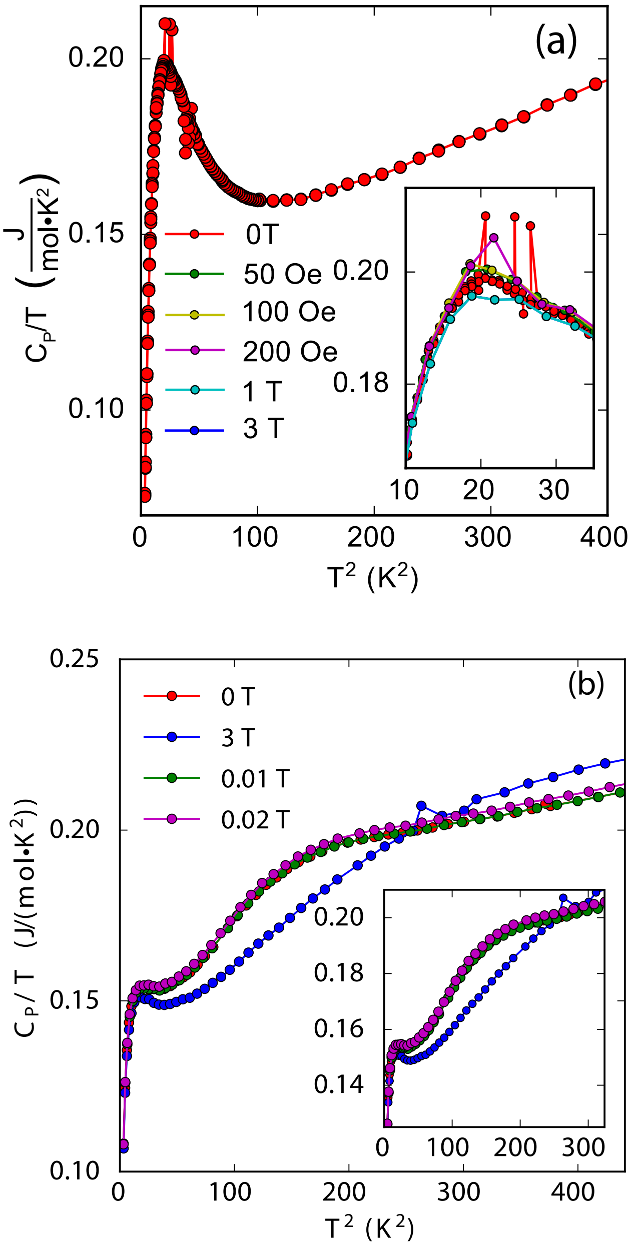

Figure S7: Specific heat measurements of (a) LiOH-intercalate FeS and (b) *inc*-Na-tochilinite.

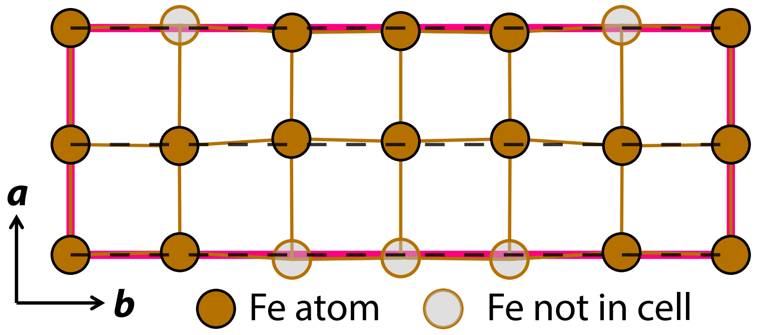

Figure S8: A projection of the Fe atoms on the (001) plane of the naturally occurring mineral tochilinite, $(2(Fe_{1-x}S)\cdot 1.8[(Mg, Fe)(OH)_2])$. A similar distortion of FeS square lattice is observed for Na-tochilinite as suggested by electron diffraction.

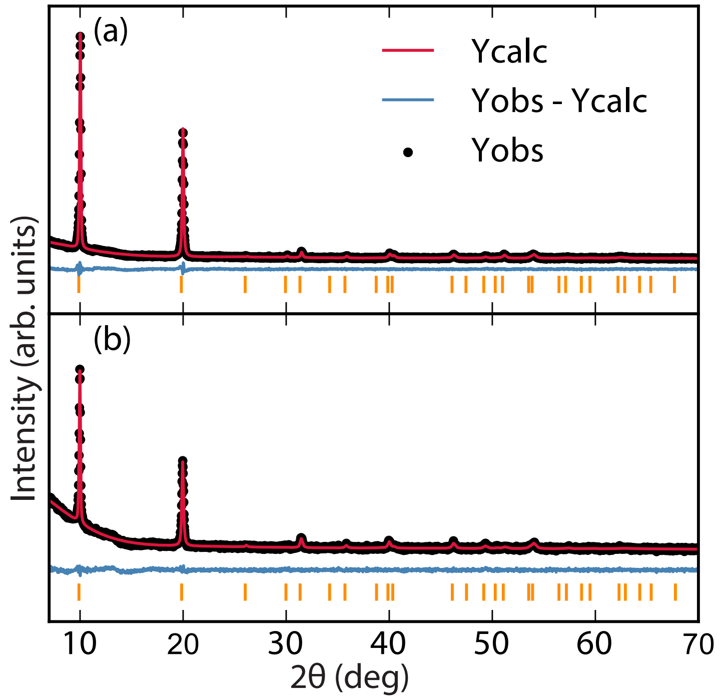

Figure S9: XRD patterns of LiOH-intercalated FeS samples shown in (a) Fig. 2a and (b) Fig. 2b, respectively. Both are fitted to a $P4/nmm$ space group and show no impurity phases.

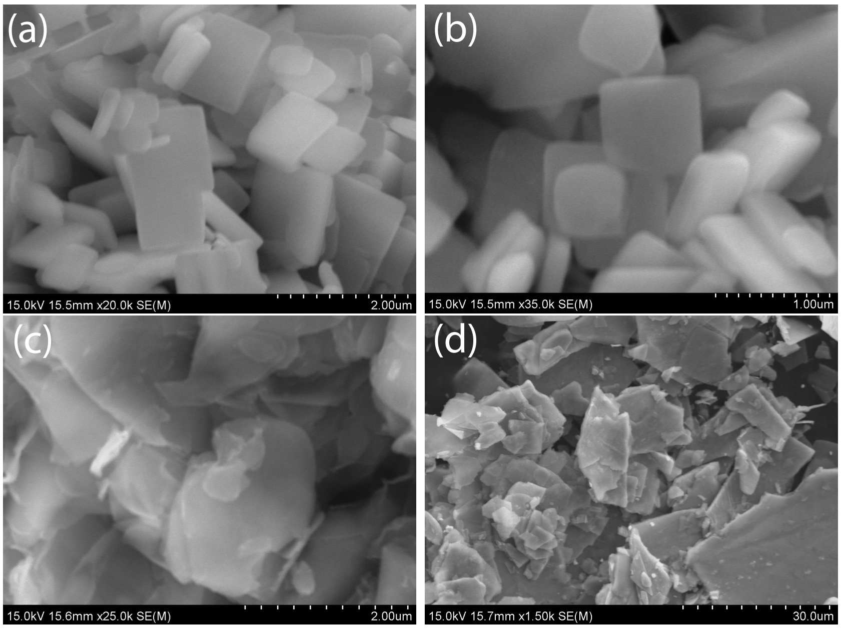

Figure S10: Scanning electron microscopy (SEM) images of (a) and (b) $(Li_{1-x}Fe_xOH)FeS$, (c) *inc*-Na-tochilinite and (d) Na-tochilinite

.
